# Weighted Phase Lag Index and Graph Analysis: Preliminary Investigation of Functional Connectivity during Resting State in Children

**DOI:** 10.1155/2012/186353

**Published:** 2012-09-24

**Authors:** Erick Ortiz, Krunoslav Stingl, Jana Münßinger, Christoph Braun, Hubert Preissl, Paolo Belardinelli

**Affiliations:** ^1^MEG Center, University of Tübingen, Germany; ^2^Center of Mind/Brain Sciences (CIMeC), University of Trento, 38068 Rovereto, Italy; ^3^Department of Cognitive and Educational Sciences (DiSCoF), University of Trento, 38068 Rovereto, Italy; ^4^Department of Obstetrics and Gynecology, University of Arkansas for Medical Sciences, Little Rock, AR 72205, USA

## Abstract

Resting state functional connectivity of MEG data was studied in 29 children (9-10 years old). The weighted phase lag index (WPLI) was employed for estimating connectivity and compared to coherence. To further evaluate the network structure, a graph analysis based on WPLI was used to determine clustering coefficient (*C*) and betweenness centrality (BC) as local coefficients as well as the characteristic path length (*L*) as a parameter for global interconnectedness. The network's modular structure was also calculated to estimate functional segregation. A seed region was identified in the central occipital area based on the power distribution at the sensor level in the alpha band. WPLI reveals a specific connectivity map different from power and coherence. BC and modularity show a strong level of connectedness in the occipital area between lateral and central sensors. *C* shows different isolated areas of occipital sensors. Globally, a network with the shortest *L* is detected in the alpha band, consistently with the local results. Our results are in agreement with findings in adults, indicating a similar functional network in children at this age in the alpha band. The integrated use of WPLI and graph analysis can help to gain a better description of resting state networks.

## 1. Introduction

Resting state networks (RSN), that is, functionally linked brain areas detectable independent of any task, are important for the understanding of brain function during development and in disease [1–8]. However, the neuronal mechanisms underlying functional connectivity at rest remain poorly understood. Recently, some limits of traditional approaches to the description of functional brain networks by means of fMRI have been reported by Power et al. [9].

We investigated the synchrony of rhythmic activity in children brain networks with magnetoencephalography (MEG). To capture the complexity of these networks, graph theory was employed on the basis of the phase-synchronization weighted phase lag index (WPLI) index. Rhythmic activity has a relevant role in the human nervous systems and has been implicated in numerous functions. Oscillatory activity in different brain areas can be synchronized (i.e., phase-coupled). Such characteristic has been hypothesized to be an important mechanism for creating an effective functional communication structure between different areas [9–13]. In this respect, the fundamental task is the application of reliable estimators to determine the phase relationship between two signals.

Here, in a first step we compared sensor connectivity mapping based on WPLI and spectral coherence which has been extensively employed for connectivity studies using MEG [10, 11, 14, 15]. Then, we used this new index to calculate graph-theoretical measures to quantify basic properties of the WPLI connectivity matrix. WPLI cannot overestimate the phase lag values due to volume conduction effects of uncorrelated noise sources. Furthermore, WPLI is less sensitive to noise than PLI and even in conditions of high signal-to-noise ratio, it shows a more reliable relationship with true phase consistency [16]. These characteristics make WPLI a suitable tool for graph analysis. We considered the MEG sensors as nodes of our graph network and the WPLI values between sensor signals as the links between the nodes.

We calculated the weighted clustering coefficient *C*, which describes local interconnectedness, and weighted path length *L*, a measure of global interconnectedness, were computed. As a measure of centrality, we calculated betweenness (BC) for each node, which is the fraction of all shortest paths in the network that pass through it. Since networks are often composed by densely interconnected groups of regions, the structure of these groups (the community structure) was also determined. Community structure consists of a separation into groups of nodes where the number of within-group links is maximized and the number of between-group links is minimized.

We tested this analysis approach with a simplified setting: by studying resting state connectivity in children, we investigated the interactions between a reference signal obtained from the peak sensor in a power cluster of channels in the occipital area and all other sensors. 

### 1.1. Resting State Connectivity from Infancy to Adult Age

We are analyzing preadolescents during resting state. Several fMRI studies have shown that the horizontal interhemispheric functional connections are already established in preadolescent children. In contrast, the anterior-posterior connections are largely reduced, compared to adults [17]. Over adolescence, short range correlation tend to weaken, whereas long-range, especially anterior-posterior connection start to strengthen [18]. The long-range connections increase over development to form complete networks like the default mode network (DMN) in adults [6, 19]. Overall, no significant connections have been found in infants between frontal and parietal areas, although the parietal area appears to contain midline and lateral parietal connections, similar to the posterior part of the DMN [20, 21]. In addition it has been shown with electroencephalography (EEG) that, between childhood and adulthood, the power in the occipital areas decreases significantly in the alpha and theta bands [22].

## 2. Methods

### 2.1. Participants

Twenty-nine children between the ages of 9 and 10 years (*M*_age_ = 9.70 years, SD ± 0.47) participated in the current study. Children who were taking any kind of medication or were diagnosed with type I diabetes, attention deficit hyperactivity disorder (ADHD) or any other chronic disease were excluded. Written informed consent was given by all children and their parents before participation. The study was approved by the Ethical Committee of the Medical Faculty of the University of Tübingen.

### 2.2. MEG Data Acquisition

Data was recorded using a 275-sensor whole-head system (VSM MedTech Ltd., Port Coquitlam, Canada). To ensure continuous recordings of the head position relative to the MEG sensors during the measurement, localization coils were attached to the nasion and the preauricular points on each side of the head of the subject. To avoid any magnetic influence from the surroundings, the MEG system was located in a magnetically shielded room (Vakuumschmelze, Germany).

The recording sampling frequency was 586 Hz. Due to technical reasons, for the present analysis, 2 of the 275 MEG sensors could not be used. Children were asked to sit quietly and relaxed with their eyes closed. Beginning and end of the 4 min resting state interval were indicated to the children by an auditory signal.

### 2.3. Data Processing

**Figure 1:**
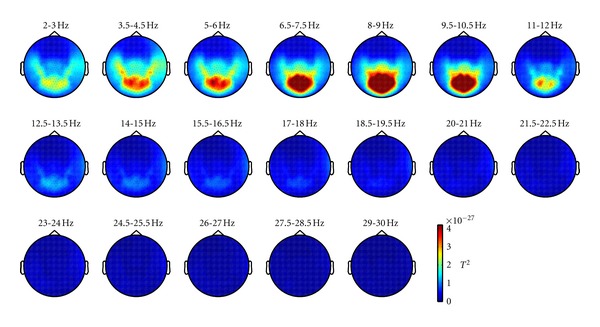
Topographic plots of sensor power in 1.5 Hz wide frequency bands, from 2 to 30 Hz. A cluster of sensors with high power is discernible in the alpha range.

Data analysis was performed using the Fieldtrip toolbox [23]. The first 20 seconds were removed from the analysis to account for initial accommodation, and the data was divided in 2-second epochs, yielding about 110 trials per dataset. A high-pass filter at 2 Hz was applied. Frequency analysis was performed in each of these trials between 2 and 30 Hz, which include the major power contribution in children. We used a multitaper smoothing of 2 Hz. The frequency range was separated in the standard frequency bands (from delta to high beta, that is, from 2 to 30 Hz, see Figure 1).

### 2.4. WPLI Description

The complex cross-spectrum *C* for two real-valued signals *x*(*t*) and *y*(*t*) is computed by Fourier-transforming them into *X*(*f*) and *Y*(*f*). Then, *X* and *Y*are used to compute the cross-spectrum *C*(*f*) = *X*(*f*)Y*(*f*), where Y* indicates the complex conjugate of *Y*. If we focus on a particular frequency of interest f*, we can consider the complex nondiagonal part of *C* as *Z*.

Then, PLI is defined as absolute value of the sign of the imaginary part of *Z*, *ℑ* [24]:

(1) $$\begin{array}{c} \text{PLI}\equiv \left|E\left\{\mathrm{sgn}\left(ℑ\left(Z\right)\right)\right\}\right|. \end{array}$$

Differently from PLI, WPLI weights the cross-spectrum according to the magnitude of the imaginary component. This allows it to limit the influence of cross-spectrum elements around the real axes which are at risk of changing their “true” sign with small noise perturbations.

Such an index of phase synchronization was proposed by Vinck and colleagues [16]:

(2) $$\begin{array}{c} \text{WPLI}\equiv \frac{\left|E\left\{ℑ\left(\text{Z}\right)\right\}\right|}{E\left\{ℑ\left(\text{Z}\right)\right\}}=\frac{\left|E\left\{\left|ℑ\left(\text{Z}\right)\right|\mathrm{sgn}\left(ℑ\left(\text{Z}\right)\right)\right\}\right|}{E\left\{\left|ℑ\left(\text{Z}\right)\right|\right\}}. \end{array}$$

This index is based only on the imaginary component of the cross-spectrum. This implies robustness to noise because uncorrelated noise sources will cause an increase of signal power. It has been shown that WPLI outperforms PLI, coherence, and imaginary coherence (IC) with real local field potentials (LFP) data [16].

### 2.5. Graph Theory Analysis

#### 2.5.1. Network Construction

In order to apply graph theory methods, a network based on WPLI functional measures was constructed. We employed an undirected weighted network [25, 26], where the nodes are the sensors, and the link weights are the WPLI values between them. 

In some cases, very weak links may obscure the topology of the significant connections. These can be removed by using a threshold, whose value is often arbitrary: therefore, a broad range of values was tested [27]. We found that most network measures were preserved over a rather broad range of threshold values.

#### 2.5.2. Network Measures

We characterized the network by using measures of segregation, integration, and centrality. For segregation, we used cluster coefficient for integration, characteristic path length, and global efficiency as well as for centrality, the betweenness centrality.

The clustering coefficient shows the fraction of the node's neighbors that are also neighbors of each other; on the other hand, betweenness of a node is defined as the fraction of all shortest paths in the network that pass through that node. The nodes with high betweenness usually bridge disparate parts in a network.

### 2.6. Community Structure and Modularity

The optimal community structure is a subdivision of the network into nonoverlapping groups of nodes in a way that maximizes the number of within-group edges, and minimizes the number of between-group edges [28, 29]. The modularity *Q* is a statistic that quantifies the degree to which the network may be subdivided into such clearly delineated groups; its values range between −1 and 1, measuring density of links inside communities as compared to links between communities.

Also, it is possible to determine a hierarchical structure of these modules [30]. Lowering the link removal threshold enabled us to reveal additional community structure.

## 3. Results

A cluster of sensors in the occipital part of the sensor array was identified as the region of interest based on the peak of the power distribution. A cut-off threshold was set at the full width at half maximum (FWHM) of the mean power spread in the alpha band (8–13 Hz). The cluster comprised 29 sensors. The signal defined by the power peak in the cluster area (central occipital sensor on the left of the midline, MLO11 in CTF MEG systems) was employed to calculate connectivity between it and the other channels (one-to-all connections).

**Figure 2:**
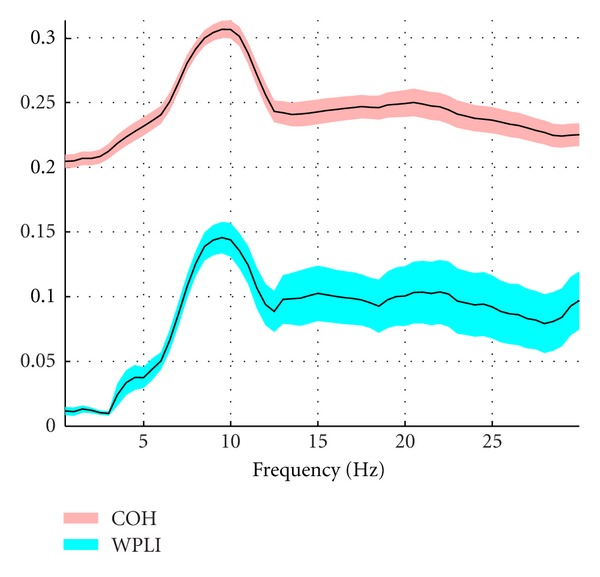
Plots of mean coherence and WPLI across subjects. Red and blue areas represent the standard error of the mean (SEM) across subjects.

Since a clear peak was shown for both connectivity measures in the range between 3 and 12 Hz (Figure 2, mean value across subjects), we focused on this range for a topographical comparison between power, coherence and WPLI. Moreover, in this range a minimum of intersubject variability is detectable (red and blue areas for coherence and WPLI, resp.).

**Figure 3:**
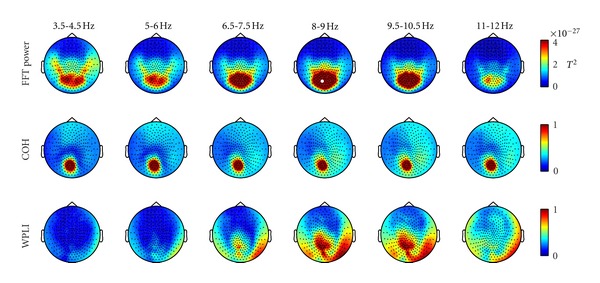
Topographic group results of sensor power, coherence, and WPLI in 1.5 Hz wide frequency bands, from 3.5 to 12 Hz. A cluster of sensors with high power is detectable in the alpha band. The sensor with the highest FFT power (MLO11, indicated by a white dot) was selected as reference node for the connectivity calculations (one-to-all connectivity). Note that the coherence topography is roughly uninformative across frequencies and strongly affected by volume conduction effects, while WPLI compares favorably in both criteria. Much more detail is discernible in the WPLI topography.

Topographically, coherence coincides well with power (Figure 3). Between 3 and 11 Hz, a single cluster of high coherence value in the occipital area was detected in coherence and power. Differently, WPLI provides a more articulated and extended connectivity map in the alpha range between 6.5 and 11 Hz. Here, an onset of different clusters (one central and two lateral ones, mostly overlapping with the power cluster) was detected in the occipital area of the sensor surface.

**Figure 4:**
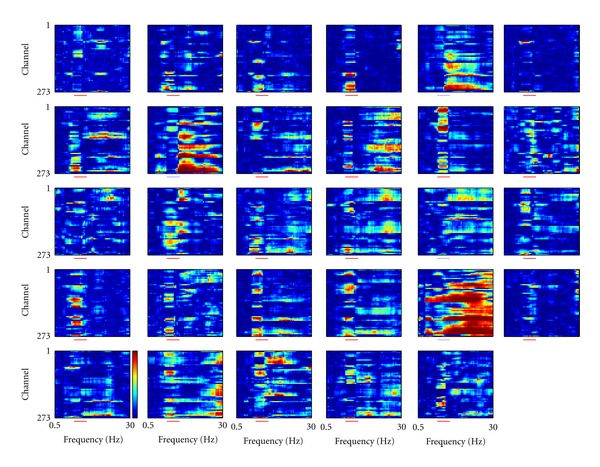
Normalized WPLI connectivity values, from/to sensor MLO11 for all frequencies. The alpha band (8 to 13 Hz) is shown by a colored bar under each plot. The color indicates whether the average WPLI in this frequency band is higher (red, 25 subjects) or lower (light blue, 4 subjects) than the average WPLI value including all frequencies.

It should be noted that the reduced variability of WPLI values in the alpha band is not due to the choice of the epoch length. Actually, this parameter showed high intersubject reliability: in 25 subjects out of 29 the channel average of WPLI values in the alpha is stronger than in the other frequency bands (Figure 4).

### 3.1. Deriving a Graph from WPLI

**Figure 5:**
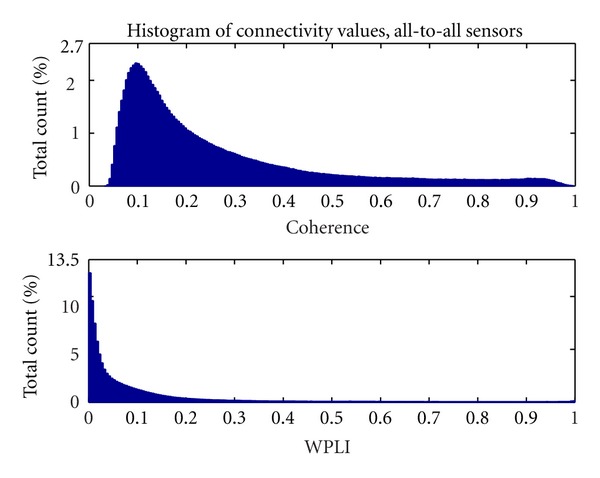
Distribution of coherence and WPLI values. Mean values across subjects are shown.

Being WPLI an inherently nonlinear measure (i.e., not proportional to either magnitude or phase of the signal frequency content), the change in the estimator value from negligible to significant values is very steep. In Figure 5, we show the distribution of values for coherence and WPLI. The WPLI distribution is relevantly steeper than the coherence distribution, representing mid-range connectivity by smaller values. Moreover, whereas coherence has non-negligible values for every combination of channels and never actually gets smaller than 0.02, WPLI has a large number of near-zero values.

The distinctions between the respective distributions are a direct consequence of the different approaches: coherence suffers from volume conduction effects, hence near-zero values will be rare. Also, WPLI is expected to be steeper, since it is a statistic on a discrete value (a sign function). Moreover, this index is weighted by the imaginary part of coherence, which is generally a minor quantity of the coherence value. As a consequence, WPLI seldom reach values near 1.

Since WPLI's values for low connectivity are very small, when assigning the weights to the network, their inverse is very high. Having fewer midrange values that map to short link lengths, good connectivity on a WPLI network is represented by comparatively longer path lengths than in a similar network drawn by coherence measures. This could be interpreted in terms of the higher specificity of the WPLI, showing fewer but more robust connections.

On constructing the network, we tested proportional thresholds for link removal from 1.0 (no links removed) to 0.1 (i.e., only 10% of links is preserved). From 1.0 to 0.3, the differences were nearly negligible; from 0.3 to 0.1, the network topology was distorted very quickly. This is not unexpected: given that WPLI tends to have a large number of near-zero values, thresholding them would make little difference indeed.

### 3.2. Local Graph Measures

**Figure 6:**
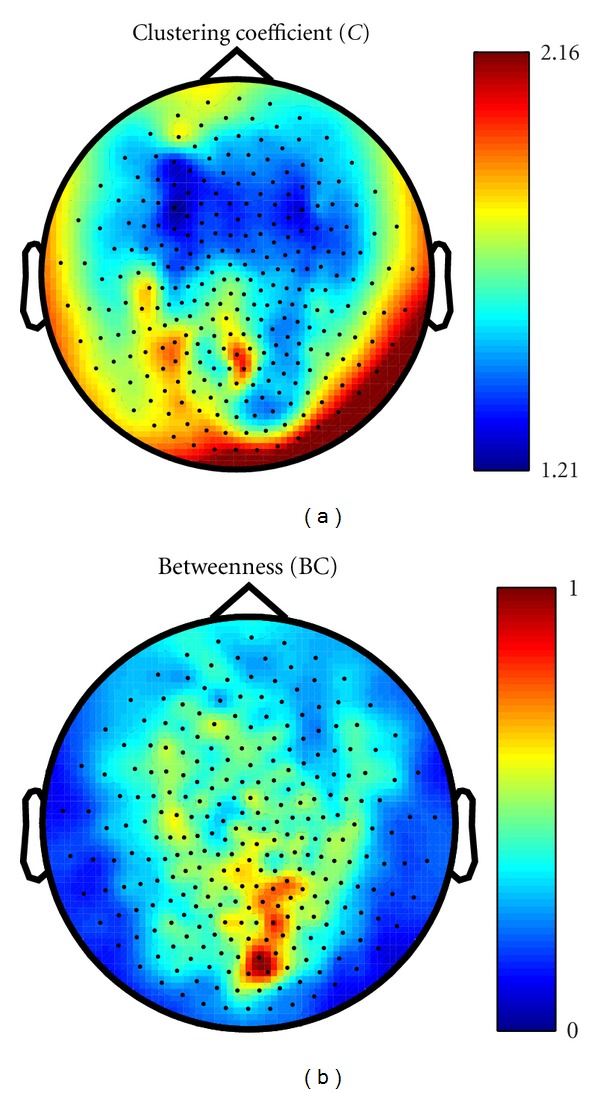
Topographic plots of graph-theoretical node-wise measures, calculated on WPLI for the alpha band, averaged across subjects. betweenness centrality BC was normalized by the plot's maximum value, while clustering coefficient was normalized by the standard deviation across subjects to minimize border artifacts.

One-to-all WPLI values were used as an input for graph theory local parameters: the clustering coefficient (*C*), betweenness centrality (BC), and modularity. Results in the alpha band show that while the cluster coefficient depicts two central and right lateral areas as separate structures, betweenness shows a clear peak on a sensor (MRO21) which is located between the areas shown by the *C* plotting (Figure 6).

### 3.3. Global Graph Measures

The characteristic path length *L* [27] was calculated as a measure of global interconnectedness based on all-to-all WPLI channel values. We considered WPLI values as weights and the mapping function as the inverse of these values. The characteristic path length was then computed for the network associated to each frequency bin, for each subject.

**Figure 7:**
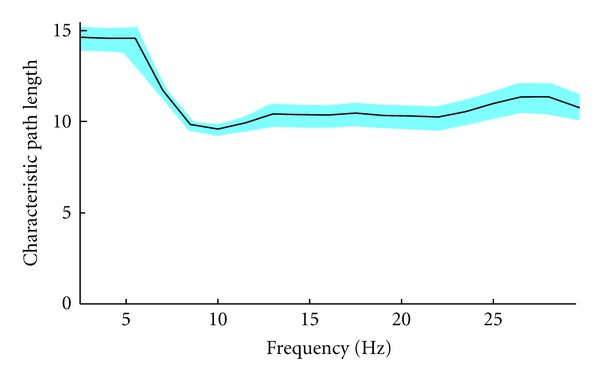
Characteristic path length, calculated on WPLI for the alpha band, averaged across subjects. The error bars show the standard error (SEM). Note how the alpha band displays not only shorter path lengths, but also smaller SEM, indicating more consistency.

The results are shown in Figure 7 over the whole investigated frequency interval.

The alpha band shows the shortest characteristic path length, although the minimum is not statistically significant.

To further investigate the relationship between sensor power and functional connectivity, we performed a linear regression between power and characteristic path length based on WPLI. The results are presented in Table 1: power does not significantly predict the characteristic path length in any band.

### 3.4. Community Structure

**Figure 8:**
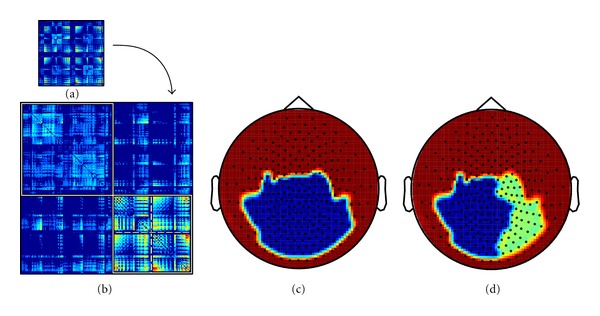
Community structure of functional connectivity in the alpha band. (a) Network connections based on WPLI, with a proportional threshold of 0.4. (b) Network reordered according to modular structure: the two top level modules are indicated by solid white lines. (c) Top level modules (*Q* = 0.2446). (d) The large occipital module can be subdivided in two smaller ones, indicated in (b) by dashed lines (*Q* = 0.2391).

Community structure was analyzed by the approach described by Blondel et al. [30], which deals efficiently with large networks and enables also to distinguish a hierarchy of modules. In a first pass, small modules are formed, and in successive passes, these are fused in larger modules while modularity *Q* increases. This is in our analysis the only case in which removing the weak links of the network led to new information. While high thresholds revealed only a large module in the occipital area, approximately corresponding to the frequency power map, we could discriminate with a threshold of 0.4 a second hierarchical level: it was composed of two smaller modules, roughly lateralized.  Figure 8 summarizes the modular structure analysis. Lowering the threshold below this level quickly degraded the analysis and did not add new information.

This hierarchy is especially relevant if integrated with the information of Figure 6(b): the nodes with highest betweenness are the ones that lie at the border of the two modules. Note that the algorithms to calculate betweenness and modularity are radically different, rendering this result more important for being revealed by two independent methods.

## 4. Discussion

The oscillatory dynamics of resting state networks in the brain is not completely understood in both children and adults [1, 31, 32]. Some recent studies employing different neuroimaging techniques have reported that children resting activity is mainly localized in the occipital lobe, with less connections to the orbitofrontal cortex than adults [20, 33, 34]. Moreover, a prevalence of low frequency (theta and alpha) oscillations has been shown in the occipital area [22]. In accordance with these findings, we found highest power between theta and alpha band (4–11 Hz). Furthermore, using a similar approach as in [1] we have used the sensor showing the peak of the power cluster as a seed for a connectivity study by means of an advanced index of phase lag synchronization (WPLI, [16]). A clear peak around 10 Hz of the mean value across subjects and channels was detected for both WPLI and coherence, roughly coinciding with a decrease of intersubject variability as measured by SEM (Figure 2).

Comparing power, coherence and WPLI (Figure 3), two relevant differences emerge between the two connectivity indices. First, while the relevant values of coherence appear to follow the power cluster as frequency increases from 3.5 to 12 Hz, the WPLI connectivity pattern is detectable exclusively in the alpha band. Secondly, a more extended and articulated picture is observable in this band in comparison to coherence and power. This is possibly due to the fact that only imaginary components of signals are considered in WPLI processing. In this way, the components propagating across close sensors with zero lag delay are discarded, avoiding overestimates of local connectivity. It can only be speculated about an analogy between the WPLI plot shown in Figure 3 and the posterior regions of the well-known default mode network [6]. It is worth noting that the local graph theory results plotted using WPLI as an input appear to be consistent with such speculation: the clustering coefficient *C*, a parameter for local segregation, shows a roughly similar pattern to the WPLI topographic plot. Moreover, a high level of betweenness was detected in an area revealed by WPLI. Betweenness quantifies the number of possible shortest paths in a network. The occipital central peak revealed in the right panel of Figure 5 (sensor MOR21) seems to confirm that this point has a relevant maximum of network connections. This result matches well with the results we obtained in an independent way for community structure (Figure 8). It is important to note that, with a small change of the modularity *Q*, the single occipital module detected in Figure 8(c) is split in two left and right modules (Figure 8(d)). The peak of betweenness lies on the border of the two modules. This set of results seems to confirm that resting state connectivity is mainly interhemispheric in preadolescent children [9].

In a future study we intend to perform a connectivity study at source level which can possibly provide further evidence of such a network. Our results agree with a previous similar study by Hillebrand and colleagues [4], showing no significant relationship between power and connectivity measures based on the imaginary part of Fourier transformed signals as WPLI (Table 1). Differently from that study, our results for the phase-lag index show a topologically different picture from power at the sensor level.

Furthermore, from all-to-all WPLI values we calculated the characteristic path length *L* of the network as an index of connectivity in all frequency bands. Consistently with [4], it was found that the alpha band showed the shortest mean path length. The strongest functional connections appear in the alpha band as the highest level of signal power does. Nevertheless, the two quantities appear to be rather independent, as shown in Table 1. This substantial agreement with adult data suggests that, despite of the evidence that the functional brain networks tend to develop from a local configuration to more distributed patterns (a path from segregation to integration) [33], several large-scale network properties are established early in development [9]. Our study used children data for the testing of different connectivity measures and associated network parameters. The current result mainly shows that the WLPI approach is justified and that our restrictive approach reveals basic network topology. It has to be stressed that this approach has to be extended to all frequency bands and applied to the source level.

## Figures and Tables

**Table 1:** *P* and *R* ^2^ values for the linear regression of power with *L* as a function of WPLI in the different frequency bands.

Frequency band (Hz)	*P* values	*R* ^ 2^
Delta (2–4)	0.8180	0.0020
Theta (4–8)	0.4675	0.0197
Alpha (8–13)	0.8675	0.0011
Low beta (13–20)	0.9501	0.0001
High beta (20–30)	0.9081	0.0005
